# Die ersten 50 roboterassistierten Donornephrektomien: „Lessons learned“

**DOI:** 10.1007/s00120-020-01302-w

**Published:** 2020-08-11

**Authors:** Philip Zeuschner, Stefan Siemer, Michael Stöckle, Matthias Saar

**Affiliations:** Klinik für Urologie und Kinderurologie, Universitätsklinikum des Saarlandes und Medizinische Fakultät der Universität des Saarlandes, Kirrberger Straße 100, 66421 Homburg/Saar, Deutschland

**Keywords:** Nierentransplantation, Nierenlebendspende, Minimal-invasive Chirurgie, Roboterassistiertes Operieren, Roboterassistierte Donornephrektomie, Kidney transplantation, Living kidney donation, Minimally-invasive surgical procedures, Robot-assisted surgery, Robot-assisted donor nephrectomy

## Abstract

**Hintergrund:**

Die minimal-invasive Donornephrektomie (DN) ist inzwischen operativer Standard, bezüglich der Rolle von roboterassistierten Verfahren gibt es bisher keinen Konsens.

**Fragestellung:**

Die ersten 50 transperitonealen roboterassistierten Donornephrektomien (RDN) einer urologischen Universitätsklinik in Deutschland wurden retrospektiv ausgewertet.

**Material und Methoden:**

Patientencharakteristika, intra- und postoperative Parameter wurden erfasst und die Nierenfunktion in einem 5‑jährigen Follow-up ausgewertet. Signifikante Prädiktoren für die Nierenfunktion bei Entlassung und ein Jahr postoperativ wurden in einem multivariablen Regressionsmodell bestimmt.

**Ergebnisse:**

Die RDN hat exzellente Ergebnisse mit niedriger Komplikationsrate, kurzer warmer (WIZ) und kalter Ischämiezeit (KIZ) sowie geringem Blutverlust und kurzer Patientenverweildauer. Die Seite der Nierenentnahme hat hierauf keine Auswirkungen. Nach RDN sind etwa 50 % der Spender formal niereninsuffizient, was aber zumeist ohne Relevanz ist, weil sich die Nierenfunktion der Spender im Follow-up nicht weiter verschlechtert. Die postoperative Nierenfunktion lässt sich bei der RDN mithilfe der präoperativen eGFR (errechnete glomeruläre Filtrationsrate) und dem Spenderalter sehr gut vorhersagen.

**Schlussfolgerungen:**

Die robotische DN stellt eine sehr gute Alternative zu anderen minimal-invasiven Operationsverfahren dar, die von Beginn an exzellente operative Ergebnisse ermöglicht.

## Abstract (en)

**Background:**

Minimally invasive donor nephrectomy (DN) is considered the gold standard, but the role of robot-assisted surgery is still controversial.

**Objectives:**

The first 50 robot-assisted DN (RDN) of a urologic transplant department in Germany were retrospectively analyzed.

**Materials and methods:**

Patient characteristics as well as intra- and postoperative surgical parameters were obtained. The kidney function of the donor was assessed within 5 years of follow-up. Predictors of postoperative kidney function at discharge and 1 year after RDN were estimated by multivariate regression analysis.

**Results:**

RDN has an excellent surgical outcome with low complication rates, short warm (WIT) and cold ischemia time (CIT), little blood loss, and short patient stay. The side of donor nephrectomy does not affect surgical outcome. After RDN, 50% of donors suffer from mild to moderate renal insufficiency without further consequences, as their kidney function does not further decrease. Preoperative eGFR (estimated glomerular filtration rate) and donor age at surgery are the best predictors of postoperative kidney function after RDN.

**Conclusions:**

Robot-assisted donor nephrectomy is an excellent alternative to other minimally invasive approaches rendering solid surgical results possible right from the start.

Die Nierenlebendspende stellt die beste Therapieoption bei terminaler Niereninsuffizienz dar. Sie stellt höchste Anforderungen an das Transplantationszentrum, nicht zuletzt deswegen, weil die Donornephrektomie an einem Gesunden erfolgt und Komplikationen schnell tragische Ausmaße haben können. Minimal-invasive Operationsverfahren nahmen 1995 mit der ersten laparoskopischen Donornephrektomie von Ratner et al. [25] Einzug in die Nierenlebendspende und führten zu steigender Akzeptanz.

Unerfreulicherweise konnten die zunehmenden Nierenlebendspenden den steigenden Bedarf an Spenderorganen jedoch nicht decken. Am 31.12.2019 standen 7148 Menschen allein in Deutschland auf der Warteliste für eine Nierenspende (vgl. Jahresbericht DSO für das Jahr 2019, s. Infobox), weltweit mehr als 120.000 Menschen.

Seit den 2000er-Jahren zählt die minimal-invasive Donornephrektomie (DN) zum operativen Standard und wird von den EAU Guidelines empfohlen [5]. Die operative Technik hat sich seit 1995 stetig fortentwickelt, auf die erste roboterassistierte DN (RDN) im Jahr 2000 folgte wenig später die erste laparoskopische Single-site-DN, bei der im LESS(„laparoendoscopic single site surgery“)-Verfahren durch nur einen Schnitt operiert wird [12]. Neben handassistierten oder retroperitoneoskopischen Varianten existieren auch solche, bei denen versucht wird, nur durch natürliche Körperöffnungen, wie z. B. transvaginal, zu operieren [24].

Insgesamt konnte gezeigt werden, dass die minimal-invasive DN der offenen DN (ODN) überlegen ist [29]. Die laparoskopische DN (LDN) hat weniger Schmerzmittelbedarf, kürzere Hospitalisationsdauer, aber längere Operations- und warme Ischämiezeiten (WIZ). Trotzdem ist die LDN hinsichtlich des perioperativen Komplikationsrisikos und Graft-Überlebens der offenen DN nicht unterlegen. Beim direkten Vergleich von LDN und RDN ist die Evidenz geringer mit bisher nur zwei prospektiven, randomisiert kontrollierten Studien [3, 20]. Es wird häufig angeführt, die RDN habe noch weniger postoperative Schmerzen und weniger Blutverlust, aber längere Operations- und WIZ [28].

Die erste RDN in Deutschland erfolgte im Jahr 2007 in der Klinik für Urologie und Kinderurologie des Universitätsklinikums Homburg. Inzwischen wurden mehr als 50 Eingriffe durchgeführt, es handelt sich um die größte Kohorte einer deutschen urologischen Klinik. In einer retrospektiven Analyse wurde das perioperative Outcome analysiert und ein Follow-up der Nierenfunktion der Spender erhoben. Signifikante Prädiktoren für die Nierenfunktion bei Entlassung und im einjährigen Follow-up wurden mittels multivariabler Regressionsanalyse bestimmt.

## Material und Methoden

Die ersten 50 RDN (2007–2019) wurden unizentrisch retrospektiv ausgewertet und ein Follow-up der Nierenfunktion erhoben. Relevante Patientencharakteristika wurden erfasst, wie das Verwandtschaftsverhältnis Spender/Empfänger. Die Seite, szintigraphische Funktion (DTPA) und Gefäßversorgung der Spenderniere wurden erhoben. Operationsdauer, Blutverlust, warme (WIZ) und kalte Ischämiezeit (KIZ), Komplikationen nach Clavien-Dindo binnen 30 Tagen sowie die Verweildauer definierten das perioperative Outcome, das hinsichtlich der Entnahmeseite verglichen wurde. Die Nierenfunktion vom Spender wurde 5–10 Jahre postoperativ erhoben, sofern möglich, und nach CKD(„chronic kidney disease“)-Stadien eingeteilt, als niereninsuffizient galt CKD-Stadium ≥3a. Signifikante Einflussfaktoren auf die Nierenfunktion und das Vorliegen einer Niereninsuffizienz wurden mittels Regressionsanalyse bestimmt.

### Operationsmethode

Die RDN erfolgten entweder mit einem DaVinci®-Si- oder -X-System (Intuitive Surgical, Sunnyvale, Kalifornien, USA). Der Patient wurde ähnlich einer transperitonealen robotischen Nierenfreilegung in Seitenlage um etwa 10–15° aufgeklappt. Die Trokare wurden pararektal von kraniokaudal in einer geraden Linie eingelegt. Bei den ersten RDN erfolgte die Nierenentnahme durch einen Pfannenstielschnitt, später durch einen GelPort®-Trokar (Applied Medical, Rancho Santa Margarita, Kalifornien, USA) periumbilikal. Dazu werden während der Operation zwei Trokare durch den GelPort® geführt, sodass die Niere nach dem Absetzen der Gefäße und Abziehen der Porthülle problemlos mit der Hand geborgen werden kann. Zur Versorgung des Nierenstils wurden zunächst Staplersysteme verwendet, aufgrund schlechter Erfahrungen später Hem-O-Lok-Clips (Teleflex Medical, Morrisville, North Carolina, USA), die in der Regel doppelt zur Aorta und V. cava hin nebeneinander auf die Gefäße gesetzt werden. Ein Abrutschen von den Gefäßen wird durch eine periphere Gefäßnaht verhindert.

### Statistische Auswertung

Kategoriale Variablen wurden in absoluter und relativer Häufigkeit angegeben, kontinuierliche als Median und Range. Fishers exakter, Mann-Whitney-U-, McNemar- und Wilcoxon-Rank-Test dienten zum Vergleich (un)verbundener Stichproben. Bei der logistischen und linearen Regressionsanalyse wurden Kovariablen nur in die multiple Regression eingeschlossen, wenn sie auch univariabel signifikant waren. Alter, präoperative eGFR (errechnete glomeruläre Filtrationsrate), eGFR bei Entlassung und Operationsdauer wurden als lineare Variablen, das Vorliegen eines arteriellen Hypertonus oder jegliche postoperative Komplikation als dichotome Variable angewendet. Die Analysen erfolgten mit SPSS v25 (IBM, Armonk, USA), alle Tests waren zweiseitig, *p* < 0,05 galt als signifikant.

## Ergebnisse

### Gesamtergebnisse

**Figure 1:**
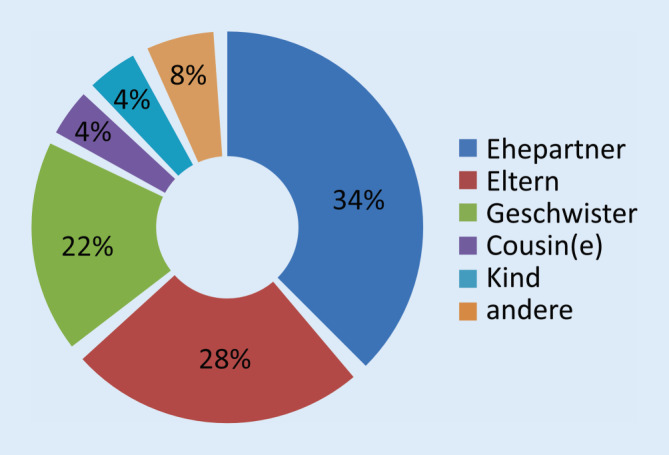

Die Nierenlebendspender waren meist Frauen im medianen Alter von 54 Jahren mit einem BMI von 25,3 kg/m^2^ (Tab. 1). Sie hatten keine relevanten Vorerkrankungen, 10 % einen Nikotinabusus in der Vorgeschichte. In den meisten Fällen erfolgte die Nierenspende unter Ehepartnern, in 14 (28 %) weiteren Fällen von Eltern an Kinder und 11 (22 %) Fällen unter Geschwistern (s. Abb. 1).

**Table 1:** 

Variable	Median (Range)/Häufigkeit
Alter (Jahre)	54 (20–69)
Geschlecht ♂	15 (30 %)
BMI (kg/m^2^)	25,3 (17,6–36,7)
*Vorerkrankungen*	
Arterielle Hypertonie	14 (28 %)
Diabetes	1 (2 %)
Nikotinabusus	5 (10 %)
*Abdominelle Voroperationen*	26 (52 %)
Appendektomie	13 (26 %)
Cholezystektomie	4 (8 %)
*Organspezifika*	
Linke Seite	40 (80 %)
Funktion	50 % (39–57 %)
*Gefäßversorgung*	
Eingefäßversorgung	42 (84 %)
Mehrere Arterien	6 (12 %)

*BMI* Body Mass Index

Die RDN dauerte im Median in 223,5 min bei einem Blutverlust von 50 ml (Tab. 2). Die warme Ischämiezeit betrug 2 min, die KIZ 76 min. Intraoperativ traten 3 Komplikationen auf, 1 (2 %) RDN wurde aufgrund massiver Adipositas und multiplen Trokardislokationen binnen der ersten 10 min nach offen konvertiert, die eigentliche DN erfolgte problemlos. In 2 (4 %) weiteren Fällen kam es zu Blutungen, einmal aufgrund einer Fehlfunktion eines Staplers, der schnitt, aber den Gefäßstumpf nicht verschloss. Diese und eine weitere Blutung aus einer Lumbalvene wurden robotisch beherrscht. Im postoperativen Verlauf trat eine Clavien-Dindo Grad 3a Komplikation auf, bei einem Ileus wurde eine Gastroskopie durchgeführt. Die mittlere Verweildauer betrug 5 Tage.

**Table 2:** 

Variable	Gesamt (*n* = 50)	Links (*n* = 40)	Rechts (*n* = 10)	*p*-Wert
Operationszeit (min)	223,5 (127–363)	223,5 (127–363)	219 (177–318)	n. s.
Blutverlust (ml)	50 (50–300)	50 (20–300)	50 (15–50)	n. s.
WIZ (min)	3 (0,5–11)	3 (1–11)	2 (0,5–3)	<0,05
KIZ (min)	76 (22–202)	76 (22–177)	117,5 (31–202)	n. s.
*Komplikationen*				
Intraoperativ	3 (6 %)	2 (5 %)	1 (10 %)	n. s.
Postoperativ	1 (2 %)	1 (2,5 %)	–	n. s.
*Clavien-Dindo*				
3a	1 (2 %)	1 (2 %)	–	n. s.
Verweildauer (Tage)	5 (2–12)	5 (3–12)	6 (2–11)	n. s.

*KIZ* kalte Ischämiezeit, *WIZ* warme Ischämiezeit

Die Entnahmeseite hatte keine Auswirkungen auf das operative Outcome, weder Operationszeit noch Komplikationsraten waren verschieden (s. Tab. 2). Lediglich die WIZ war bei den 10 (20 %) rechtsseitigen Nierenentnahmen kürzer (2 vs. 3 min, *p* < 0,05).

### Follow-up der Nierenfunktion

**Figure 2:**
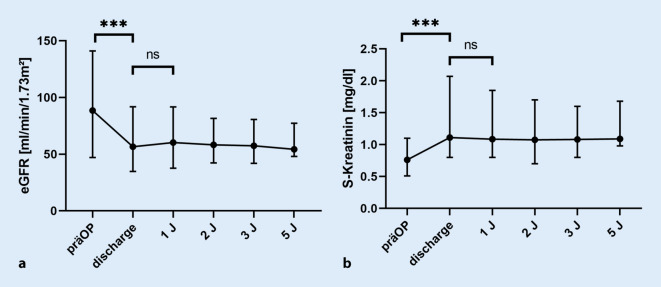

Zum Zeitpunkt der Entlassung war die Nierenfunktion des Spenders signifikant schlechter (eGFR 88,5 vs. 56,6; *p* < 0,001), danach blieb sie unverändert (Abb. 2). Vor der Transplantation war kein Patient niereninsuffizient, nach RDN hatten fast die Hälfte ein CKD-Stadium ≥3a (*p* < 0,001, Tab. 3). Der Anteil niereninsuffizienter Patienten nahm in den Folgejahren tendenziell zu, wobei sich die Nierenfunktion nicht signifikant verschlechterte und erst bei 2 Patienten die RDN 10 Jahre zurückliegt.

**Figure 3:**
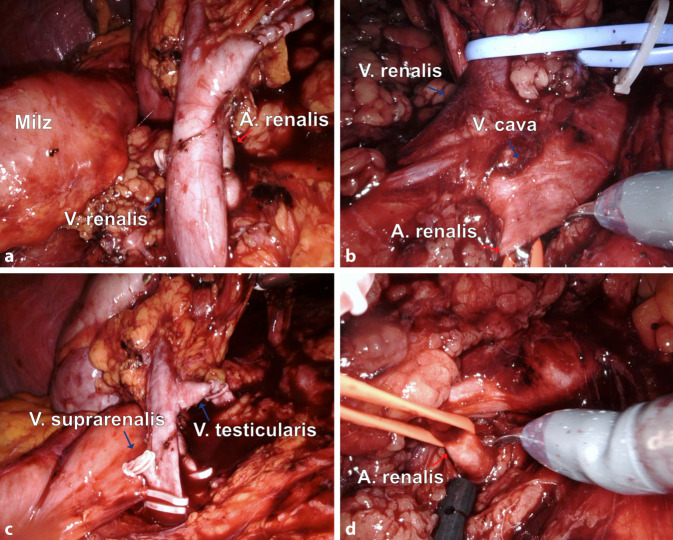

**Table 3:** 

CKD-Grad	eGFR	Präoperativ	Postoperativ	1 J	2 J	3 J	5 J	10 J
1	≥90	48 %	2 %	4 %	–	–	–	–
2	60–90	53 %	32 %	48 %	44 %	33 %	–	100 %
3a	45–59	–	46 %	33 %	50 %	62 %	27 %	–
3b	30–44	–	2 %	15 %	6 %	5 %	73 %	–
4	15–29	–	–	–	–	–	–	–
5	<15	–	–	–	–	–	–	–
*n* =	–	50	50	27	18	21	11	2

*CKD* „chronic kidney disease“, *eGFR* errechnete glomeruläre Filtrationsrate

In der multivariablen Regression war die präoperative Nierenfunktion sowohl für die postoperative Nierenfunktion (B-Wert 0,51, *p* < 0,001) und das Vorliegen einer Niereninsuffizienz prädiktiv (OR 0,9, *p* < 0,01, Tab. 4). Ein höheres Spenderalter sowie das Vorliegen eines Hypertonus waren nur univariabel signifikant. Operative Parameter hatten *keinen* Einfluss auf die Nierenfunktion zum Zeitpunkt der Entlassung.

**Table 4:** 

	**eGFR Entlassung**		**Niereninsuffizienz Entlassung**	
	*B‑Wert (95* *%-KI)*	*p‑Wert*	*OR (95* *%-KI)*	*p‑Wert*
Alter	–	n. s.	–	–
Hypertonus	–	n. s.	–	n. s. (0,07)
eGFR präoperativ	0,51 (0,31; 0,71)	<0,001	0,9 (0,86; 0,97)	<0,01
Operationsdauer	–	–	–	–
Komplikationen	–	–	–	–
	**eGFR 1 J**		**Niereninsuffizienz 1 J**	
	*B‑Wert (95* *% KI)*	*p‑Wert*	*OR (95* *% KI)*	*p‑Wert*
Alter	−0,43 (−0,7; −0,16)	<0,05		n. s.
Hypertonus	–	n. s.	–	–
eGFR präoperativ	0,51 (0,21; 0,8)	<0,01	0,72 (0,53; 0,98)	<0,05
eGFR Entlassung	–	n. s.	–	n. s.
Operationsdauer	–	–	–	–
Komplikationen	–	–	–	–

*KI* Konfidenzintervall, *eGFR* errechnete glomeruläre Filtrationsrate, *OR* Odds Ratio

Bezüglich der einjährigen Nierenfunktion war nicht nur die präoperative Nierenfunktion (B-Wert 0,51, *p* < 0,01), sondern auch das Lebensalter (B-Wert −0,43, *p* < 0,05) multivariabel signifikant (Tab. 4). Das präoperative Vorliegen einer arteriellen Hypertonie sowie die postoperative Nierenfunktion waren nur univariabel prädiktiv. Für das Vorliegen einer Niereninsuffizienz 1 Jahr nach Operation war ausschließlich die präoperative Nierenfunktion ein Prädiktor (OR 0,72, *p* < 0,05).

## Diskussion

In dieser Studie wurden retrospektiv die ersten 50 robotischen Donornephrektomien (RDN) eines urologischen Zentrums in Deutschland ausgewertet, wobei die erste RDN im Juni 2007 erfolgte. Das oberste Ziel einer DN ist die Sicherheit des Donors, da sich ein überdurchschnittlich Gesunder einem elektiven Eingriff in altruistischer Intention unterzieht. Die Komplikationsrate der ersten 50 RDN war sehr gering mit 3 (6 %) intraoperativen Komplikationen inklusive einer Konversion nach offen wegen multipler Trokardislokationen bei Adipositas. Die häufigsten Komplikationen bei RDN aber auch der LDN sind Blutungen [7]. Auch hier traten zwei Blutungen auf, eine aus der Lumbalvene und eine aus der A. renalis infolge eines defekten Staplersystems. Beide Blutungen konnten robotisch kontrolliert werden und führten nicht zu Massenblutungen.

Die Versorgung des Gefäßstils bei der Donornephrektomie ist seit einer Warnung der FDA zum Einsatz von Hem-O-Lok-Clips bei der LDN im Jahr 2006 v. a. aus rechtlicher Sicht komplex geworden (Abb. 3; [1]). Zuvor waren bei zwei Nierenspendern insuffizient schließende Clips abgerutscht, und die Patienten verbluteten [8]. Die FDA und auch die Deutsche Transplantationsgesellschaft warnten deshalb vor der Verwendung von Hem-O-Lok-Clips bei der LDN. Wir änderten unser Vorgehen und setzen fortan bei jeglicher roboterassistierten Nephrektomie Staplersysteme ein, jedoch wie in anderen Kliniken mit erheblichen Problemen aufgrund vieler Fehlfunktionen, auch bei einer RDN [4]. Aus diesem Grund wurde sich dazu entschlossen, unter gewissen Vorsichtsmaßnahmen doch wieder Clips zu verwenden. Entscheidend ist wahrscheinlich, dass der Clip nicht direkt an der Aortenwand platziert wird, da ihn dort die kontinuierliche Gefäßpulsation zum Abrutschen bringt. Aus dem gleichen Grund sollte die Arterie nicht direkt am Clip, sondern mit einem Sicherheitsabstand von 2–3 mm abgetrennt werden. Dann ist das Risiko nicht größer als bei einer gewöhnlichen Nephrektomie, insbesondere rechtsseitig, wo regelhaft ein Stück weit entfernt von der Aortenwand abgesetzt wird. Als weitere Vorsichtsmaßnahme setzen wir zusätzlich einen zweiten Clip weiter peripher, eine Durchstechungsligatur dient als Abrutschsicherung. Auf diese Art und Weise haben wir bis heute keine negativen Erfahrungen bei der Verwendung von Hem-O-Lok-Clips gemacht. Eine Metaanalyse konnte zudem *keine* Unterschiede zwischen Staplern und Clips hinsichtlich der Blutungskomplikations- oder Todesrate zeigen [19]. Alternativ kann auch auf Titanclips zurückgegriffen werden. Bei der Verwendung von Hem-O-Lok-Clips sollten Nierenspender jedoch einer gesonderten Risikoaufklärung unterzogen werden, dies ist auch in anderen deutschen Transplantationszentren der Fall [6]. Ob eine gesonderte Risikoaufklärung in diesem Spannungsfeld auch zukünftig rechtlich haltbar ist, wird sich zeigen. Insofern sollte jedes Zentrum, das Donornephrektomien durchführt, für sich selbst entscheiden, welche Methode zur Gefäßkontrolle die Beste ist.

### Lesson #1: Die Verwendung von Hem-O-Lok-Clips sollte nur mit Bedacht erfolgen

Die Einführung minimal-invasiver Operationstechniken bei der DN führte weltweit zunächst zu weiteren Problemen. Metaanalysen zeigten, dass bei der LDN rechtsseitige Nieren aufgrund ihrer komplexeren Gefäßanatomie bei der Lebendspende signifikant häufiger eine delayed graft function (DGF), höhere Graft-Thromboserate sowie insgesamt ein schlechteres Graftüberleben als linke Nieren hatten [15, 18]. Diese Unterschiede waren gering und in spezialisierten Zentren (fast) nicht messbar, weswegen die EAU Guidelines bei der LDN heute von einer gleichwertigen Sicherheit im Vergleich zur ODN ausgehen [5]. Das Outcome der ersten 50 RDN zeigt im Seitenvergleich keine signifikanten Unterschiede (Tab. 2), lediglich die WIZ war bei den linksseitigen Nierenentnahmen geringfügig länger (3 vs. 2 min, *p* < 0,05). Dies ist mit einem Lernkurveneffekt zu erklären, denn rechtsseitige Nieren wurden erst bei steigender Erfahrung mit der RDN durchgeführt. Dass sich die restlichen Parameter nicht unterscheiden, ist am ehesten darauf zurückzuführen, dass sich die Vorzüge des robotischen Operierens im Vergleich zur Laparoskopie besonders in komplexen Situationen zeigt: bestimmte Manöver verlangen dem Operateur beim Laparoskopieren deutlich mehr Geschick ab als am Roboter – unsere Blutungskomplikationen mussten so nicht konvertiert werden. Die Studienlage ist diesbezüglich nicht eindeutig, einige Arbeiten zeigen Unterschiede zwischen rechts- und linksseitiger RDN, andere nicht [6, 26]. Aus rein operativer Sicht spielt die Organseite für die RDN unserer Erfahrung nach eine untergeordnete Rolle, vielmehr sollten Nierenfunktion oder anatomische Besonderheiten für die Seitenwahl maßgeblich sein. Der Operateur sollte jedoch im Zweifelsfall die operative Technik den Gegebenheiten anpassen und mehrere beherrschen, von ODN über LDN oder auch die RDN.

### Lesson #2: Für die RDN ist die Organseite unerheblich

Die mediane Operationszeit von 223,5 min ist im Vergleich zu anderen Arbeiten etwa 30–60 min länger [28]. Mehrere Gruppen beschreiben bei der RDN eine Lernkurve, da die Operationszeit mit steigender Erfahrung abnimmt [13]. Ganz unabhängig davon sind die Operationszeiten bei Horgan et al. [13] schon bei den ersten Fällen kürzer (201 ± 42 min), denn nicht mangelnde Erfahrung, sondern die Abläufe im Universitätsklinikum Homburg führen zu einer längeren Operationszeit. Um eine kurze KIZ zu ermöglichen, werden Spender und Empfänger simultan in zwei Sälen von zwei Teams operiert. Die Niere wird erst dann abgesetzt, wenn das Transplantationsteam sicher ist, dass die Transplantation wie geplant möglich ist. Dies kann dazu führen, dass ein Team auf das andere warten muss, was die Operationszeit verlängert, aber die KIZ signifikant verkürzt. Die französischen Kollegen aus Nancy erreichen nach 150 RDN eine mediane Operationszeit von 176 ± 23 min, aber nehmen eine fast dreimal so lange KIZ von 206 ± 71 min in Kauf, weil sie die Patienten nacheinander operieren [17]. Auch wenn eine längere KIZ kein signifikanter Prädiktor für eine postoperativ schlechtere Nierenfunktion ist, zeigt sich tendenziell ein schlechteres Outcome, wenn die KIZ 2 h übersteigt [9, 22]. Während der „Wartezeit“ auf das Absetzen der Niere wird das Pneumoperitoneum bei uns vollständig abgelassen, damit sich die Spenderniere erholen kann. In verschiedenen Arbeiten konnte gezeigt werden, dass das Pneumoperitoneum negative Auswirkungen auf die Mikrozirkulation hat und daher die Niere(n) schädigen kann [23, 27]. Möglicherweise genau infolge dieser „Erholungspause“ setzt in den meisten Fällen bereits intraoperativ die Urinproduktion bei der Transplantation ein, die seit 2016 auch robotisch durchgeführt wird [30]. Um den Empfängern optimale Voraussetzungen für eine bestmögliche Nierenfunktion zu bieten, werden wir zukünftig an dieser Vorgehensweise festhalten.

### Lesson #3: Sorgfalt geht vor

Häufig wird auch gegen die RDN angeführt, die WIZ sei im Vergleich zur LDN länger, sie beträgt hier 3 min (Tab. 2; [3]). Dies ist damit zu erklären, dass bei der RDN der Operationsroboter vom Situs abgedockt werden muss, beim laparoskopischen Vorgehen nicht. Wir verwenden schon seit Längerem einen GelPort®-Trokar, dessen Kappe problemlos und schnell entfernt werden kann. Ganz unabhängig davon ist es sehr unwahrscheinlich, dass kleine Unterschiede von nur wenigen Minuten WIZ überhaupt Auswirkungen auf die postoperative Nierenfunktion haben. In der Nierentumorchirurgie konnte gezeigt werden, dass eine WIZ von unter 20 min nur eine geringe Auswirkung auf das Organ hat [10]. Auch für die Nierentransplantation wurde belegt, dass erst eine WIZ >45 min die Organfunktion bei Nierenlebendspenden signifikant verschlechtert, darunter konnten keine negativen Auswirkungen festgestellt werden [11]. Insofern ist eine WIZ von 2–3 min ein exzellentes operatives Ergebnis.

### Lesson #4: Die WIZ ist auch bei RDN kurz

Die postoperative Nierenfunktion des Spenders ist im Vergleich zur präoperativen Situation schlechter (Abb. 2). Im Follow-up nahm die Nierenfunktion nicht weiter ab, aber verbesserte sich auch nicht mehr. Damit sind fast 50 % der Nierenspender nach RDN formell niereninsuffizient. Auch wenn ehemals Arbeiten titelten, die Nierenfunktion von Nierenlebendspendern sei im Vergleich zur Allgemeinbevölkerung langfristig gleich, so trifft dies in der Realität nicht zu [14]. Das Risiko für terminale Niereninsuffizienz ist in gematchten Kollektiven *erhöht*, weil Nierenspender gesünder sind als die Normalbevölkerung – trotzdem ist das Risiko immer noch als gering einzuschätzen [21]. Im eigenen Kollektiv ist bisher kein einziger Patient niereninsuffizient geworden, die Nierenfunktion der Spender hat sich seit der RDN während des Follow-ups auch nicht verschlechtert (Abb. 2).

In einer Regressionsanalyse sind Patientenalter, arterielle Hypertonie und präoperative Nierenfunktion gute Prädiktoren für die Nierenfunktion bei Entlassung und im einjährigen Follow-up nach RDN (Tab. 4). In der multivariablen Regression war jedoch nur noch die präoperative Nierenfunktion signifikant, bei der einjährigen eGFR auch das Alter (B-Wert −0,43, *p* < 0,05). In diesem Zusammenhang entwickelten Benoit et al. [2] 2017 ein Vorhersagemodell für die postoperative Nierenfunktion ein Jahr nach LDN [16]. Dieses Modell lässt sich sehr gut auf die RDN übertragen, weil bei Benoit et al. [2] ebenso nur präoperative eGFR und das Donoralter Prädiktoren für die einjährige eGFR sind. Erstaunlicherweise unterscheiden sich die Koeffizienten nur gering: bei der RDN errechnet sich die eGFR ein Jahr postoperativ mit 32,29 + (0,56 × präoperative eGFR) − (0,44 × Alter), bei der LDN nach Benoit et al. mit 31,17 + (0,52 × präoperative eGFR) − (0,31 × Alter). Da operative Parameter auch bei der RDN keinen Einfluss auf die postoperative Nierenfunktion haben, ist das Operationsverfahren unwichtig.

### Lesson #5: Präoperative Nierenfunktion und Donoralter zählen

Diese Arbeit ist nicht frei von Limitationen. Es handelt sich zwar um die größte monozentrische Kohorte einer urologischen deutschen Transplantationsklinik, jedoch spiegelt sie nur eine Perspektive wider, zumal am Universitätsklinikum Homburg fast keine laparoskopischen Eingriffe durchgeführt werden. Es handelt sich somit um Ergebnisse eines großen robotischen Zentrums, die nicht zwangsläufig auf jede Klinik übertragbar sein müssen.

#### Infobox Mehr Informationen zum Thema

- DSO-Jahresbericht: https://tinyurl.com/y73h8bc6
- DDVZ: https://tinyurl.com/yde4hjp4
- AK NTx: https://www.nieren-transplantation.com/

## Fazit für die Praxis

- Die robotische Donornephrektomie (RDN) hat sich mit weltweit mehr als 1000 Eingriffen als operatives Standardverfahren in spezialisierten Zentren etabliert.
- Die ersten 50 RDN an einem deutschen urologischen Zentrum haben exzellente Ergebnisse mit kurzer WIZ (warme Ischämiezeit) und KIZ (kalte Ischämiezeit).
- Die RDN stellt wie die laparoskopische DN (LDN) besondere Anforderungen hinsichtlich der Versorgung der Nierengefäße.
- Die rechtsseitige RDN hat im Gegensatz zu LDN-Serien, die anfänglich rechts schlechtere Ergebnisse als links zeigten, von Beginn an ein gleichwertiges operatives Outcome.
- Selbst wenn die RDN technisch womöglich einfacher als die LDN ist, steht sie vor denselben komplexen organisatorischen Anforderungen im Spannungsfeld medizinscher, ethischer aber auch ökonomischer Faktoren.
- Ein hoher Anteil an Nierenspendern ist nach RDN formell niereninsuffizient, wobei präoperative Nierenfunktion und Lebensalter hierfür maßgeblich sind – dies hat in der Praxis zumeist keine große Relevanz.

## Abkürzungen

CKD: „Chronic kidney disease“
DN: Donornephrektomie
KIZ: Kalte Ischämiezeit
LDN: Laparoskopische Donornephrektomie
ODN: Offene Donornephrektomie
RDN: Roboterassistierte Donornephrektomie
WIZ: Warme Ischämiezeit
